# Enhancing quality and integrity in biomedical research in Africa: an international call for greater focus, investment and standardisation in capacity strengthening for frontline staff

**DOI:** 10.1186/s12910-015-0071-3

**Published:** 2015-11-13

**Authors:** Participants of an International Workshop in Kenya on the Role of Frontline Staff in Biomedical Research, July 2014, Francis Kombe

**Affiliations:** KEMRI/Wellcome Trust Research Programme, Kilifi, Kenya

**Keywords:** Africa, Continuing professional development (CPD), Fieldworkers, Health Research Ethics

## Abstract

The integrity of biomedical research depends heavily on the quality of research data collected. In turn, data quality depends on processes of data collection, a task undertaken by frontline research staff in many research programmes in Africa and elsewhere. These frontline research staff often have additional responsibilities including translating and communicating research in local languages, seeking informed consent for study participation and maintaining supportive relationships between research institutions and study participants and wider communities. The level of skills that fieldworkers need to undertake these responsibilities clearly affects the quality of data collected, the ethics of research ‘on the ground’ and the short and long term acceptability of research.

We organised an international workshop in Kenya in July 2014 to discuss the role of frontline staff in scientific research. A total of 25 field managers from 9 African countries and the UK met for 2.5 days to discuss the relationship between data quality and institutional performance management systems and how they affect career progression and supportive supervision policies of research frontline staff.

From this workshop, and supporting an expanding literature on the role of fieldworkers in international health research, participants agreed that fieldworkers’ roles present them with practical and ethical challenges that their routine training does not adequately prepare them for. We argue that the common and complex challenges facing fieldworkers should in part be addressed through increased investment and collaborative agreements across types of research institutions in Africa. We call for standardization of core elements of training for this critically important cadre of research staff who perform similar roles and encounter similar challenges in many African settings. Although many valuable training elements are offered in institutions, there is a need to develop broader, more grounded and innovative strategies to address complex realities for fieldworkers, and support the integrity and ethics of health research in these settings.

## Introduction

The integrity of biomedical research depends heavily on the quality of research data collected [1]. In turn, data quality depends on processes of data collection, a task undertaken by frontline research staff in many research programmes in Africa and elsewhere. These frontline research staff, often known as fieldworkers, frequently have additional responsibilities including translating and communicating research in local languages, seeking informed consent for study participation and maintaining supportive relationships between research institutions and study participants and wider communities [2–5]. The level of skills fieldworkers require to undertake these responsibilities clearly affects the quality of data collected, the ethics of research ‘on the ground’ and the short and long term acceptability of research [6].

In this article, we argue that there is a pressing need to strengthen the capacity of fieldworkers to support high quality ethical research in Africa. Drawing on an initiative involving research institutions from nine African countries, including a survey and international workshop on the roles of fieldworkers in research in Africa, we argue for increased investment and collaborative agreements across institutions to address the challenges that fieldworkers face. Through these recommendations, we aim to highlight the roles of fieldworkers in health research in Africa and elsewhere, and stimulate debate on how high standards of fieldworker practice can be achieved.

## Background to the workshop

In July 2014, an international workshop on fieldworkers’ roles and institutional capacity building in research centres in Africa was held in Kilifi, Kenya, with support from the Global Health Bioethics Network [7]. The workshop was preceded by a telephone survey of 20 African research centres to map out existing performance management systems in these institutions. Centres included in the survey were identified through membership of the INDEPTH Network [8], an international research network for health and demographic surveillance sites in Africa, or through networks established by the Kenya Medical Research Institute/Wellcome Trust Research Programme (KWTRP) training department.

At the workshop, experienced fieldworker managers from 15 research centres employing 2136 fieldworkers across 9 countries shared experiences of capacity strengthening activities. The participants ‘represented’ a total workforce of 2136 fieldworkers within these institutions. Other workshop participants were senior managers of the INDEPTH Network and social scientists from Kenya and the Gambia. A particular skill set was contributed by a social scientist at the UK Medical Research Council working on fieldworker training modalities combining on-line and face-to-face components (or ‘blended learning’) at the MRC Laboratories in the Gambia (see full list of participants at the end of this article). Fieldworker managers were included as a group able to reflect on different aspects of fieldworker performance from experience. Several managers had themselves worked as fieldworkers at earlier points in their careers and were also able to represent this perspective directly.

## Workshop activities

Over two and a half days, participants at the workshop were involved in a range of information-sharing and deliberative activities towards sharing experiences and building consensus on the most important and achievable strategies needed in the area of fieldworker capacity building and performance management. Three experienced facilitators supported activities, including plenary presentations, small and plenary discussions, and participatory consensus building activities (such as round table discussions, involving the movement of small groups around tables allocated to different topics, identifying and expanding core arguments and recommendations in turn). The main workshop topics are summarized in Table 1 below:

**Table 1:** Topics for discussion at the workshop

*Workshop presentations included:*
• Feedback on the findings of the telephone survey
• Descriptions of fieldworkers’ roles, facilitating factors and challenges in participating sites
• Key findings from recent social science research on fieldworkers’ roles
*Workshop discussion topics included:*
• What is an ‘ideal’ fieldworker: What generic and specific activities (roles) do fieldworkers carry out, what skills do they need to undertake their roles ethically and effectively and how do they acquire these key skills?
• What is the optimal support needed for fieldworkers to carry out their roles effectively? In areas of skills building and enhancement; structural support within institutions; and community engagement
• What areas can we influence and what does this require? (round table discussion)
• Joining the loose ends: Similarities and differences in ways forward for centres (plenary discussion)

All workshop proceedings and discussions were documented by a dedicated note taker, supplemented by transcriptions of ‘flipchart notes’ generated by small and plenary group discussions. A report was developed from these outputs, led by the main facilitators and supported by a core writing group identified during the workshop. The report was finalized after feedback and agreement from all participants had been incorporated.

## Issues discussed and emerging during the workshop

### Fieldworker’s roles and training needs

Fieldworkers in these centres are a diverse group of staff, but a majority are secondary school leavers recruited from geographic communities in which studies are conducted. Fieldworkers typically undertake a range of frontline activities to support studies, including seeking informed consent for study participation, collecting different types of data and undertaking non-invasive sample-taking or measurement procedures. All fieldworkers in these centres are routinely trained on study specific techniques, but managers universally recognised the practical and ethical challenges and dilemmas presented by the ‘expanded roles’ of fieldworkers; building relationships, responding to community members’ expectations and needs, and supporting both community understanding of research and recruitment into studies [3, 5, 9, 10]. There was recognition that these are complex roles that include balancing of institutional roles and guidelines against the community expectations and fieldworkers’ personal interests. In this way, fieldworkers continuously make independent (often unconscious) decisions on how to apply ‘ethical principles’ in reality. Examples of the ethical challenges fieldworkers were reported to face include dealing with silent refusals [11] (participants who may not openly refuse to participate in research but indirectly avoid key study procedures), dealing with increasing demands for study benefits from community members who may or not necessarily be study participants and how to deal with study participants who may genuinely be in need of support which the study cannot offer [9, 12, 13].

In addition, fieldworkers are commonly seen by community members as the face of research institutions, and by researchers as reflecting community positions. They have to balance if and how to follow research or employer’s guidelines when these differ from cultural norms. [13, 14] In doing this, they play a central role within some of the poorest communities in rural and urban Africa as the frontline of often very well resourced international research institutions. This may create tensions in understanding researchers’ and their own responsibilities as they respond to important unmet needs within communities, and how this should be done. In doing so, their positions reflect a different and wider ‘frontline’ generated by global forms of structural inequity increasingly recognised as an important ethical issue in international health research [15, 16].

### Recognising challenges in current training activities

A series of challenges around common training practices were identified, including:

- Many fieldworkers’ training curricula focus on technical skills such as phlebotomy, measurement of anthropometric and vital signs, interviewing and Good Clinical Practice, but do not sufficiently support development of ‘soft’ skills or understanding of the ethical dimensions of these challenges commonly met at work [6].
- In research, fieldworker training activities are often developed in response to specific project needs, and lack a standardized approach in relation to important cross-cutting skills (importantly including the ‘soft skills’ described in the previous paragraph). For example, the existence of differing types and levels of skills has the potential to generate differences in the data collected. Responding to community stakeholders’ questions and requests for support by providing different information and in different ways can cause confusion and generate negative attitudes and rumours within ‘study communities’. Increasing the extent to which training in these areas are more standardized, for training within and across institutions within Africa, would promote wider use of these core skills and build recognition of their importance.
- Fieldworkers, like other cadres of staff, hope to grow professionally with time but many institutions do not support fieldworker career development. Poorly defined career paths demotivate fieldworkers, who commonly see themselves ‘stuck at the bottom of the pile’ in research institutions. Since this group of staff is central to the integrity of research, there is a fundamental mismatch between perception and reality in relation to fieldworkers’ contributions to research.
- Increasing amounts of research in Africa [17] and increasing technological advances in research increase the complexity of studies that fieldworkers should understand and communicate to study participants. Methods for data collection used by fieldworkers are also becoming increasingly technology-driven, requiring the use of mobile phones, tablets and palm-held devices. These and other developments ratchet up the need for technical knowledge and skills within this staff group.

## Recommendations from the workshop

Many of the issues for fieldworker capacity building were shared by all sites represented at the workshop, and are reflected in the literature [6, 9, 18]. On the basis of these discussions, workshop participants put forward three areas of recommendations to address cross cutting challenges in fieldworker capacity building and strengthen the future of biomedical research in Africa:

1. *Increase institutional recognition of fieldworker’s roles and the need for systematic and comprehensive capacity building*
  Research institutions should appreciate the expanded roles fieldworkers play in supporting high quality biomedical research, and the complex practical and ethical challenges they face. This should be reflected in fieldworkers’ job descriptions, performance appraisals and capacity building activities. Capacity building should include training and forms of regular supportive supervision that provide opportunities for challenges experienced in practice to be discussed openly and resolved with supervisors. To ensure that adequate resources are allocated, capacity building activities should be described in research proposals and grant applications. A similar form of institutional ‘buy-in’ has already occurred internationally for highly related processes of community engagement, following funders’ requirements that researchers include these descriptions in research protocols.
2. *Develop common areas of a core curriculum for field worker capacity building to enhance quality of training processes*
  Noting that fieldworkers in different settings often have very similar roles, research institutions should collaborate to develop common areas of training curricula that build on strengths and experiences across all centres. A particular importance of this form of collaboration is in supporting multi-site studies that are increasingly common within Africa. Common areas for training could include knowledge about basic biology, research approaches and methods, research ethics and research regulatory frameworks. Common skills include those for data collection and documentation and – critically – ‘soft’ skills such as respectful communication, and being aware of and managing ethical challenges and issues in practice. At the same time, some flexibility across such a joint curriculum would be important given differences in context across sites.
3. *Increase emphasis on fieldworkers’ career development including developing regionally accredited training*
  As well as taking forward existing learning about such processes, building a joint core curriculum offers potential to support fieldworkers’ professional development through establishing regionally accredited training processes. Regionally accredited training would increase individual motivation and enhance capacity for employment across different research organizations, acting as a form of empowerment. From a researchers’ perspective this may enable prompt recruitment, reduce the time spent in training newly employed fieldworkers, and address concerns associated with losing skilled workers at the end of short-term projects. At an institutional level, centres could optimise use of resources for fieldworkers’ professional development by pooling these between different projects or running such an initiative centrally.
  Biomedical research does not stand still. Similarly there is a need to continually upgrade fieldworkers’ training to meet new technological requirements. Research institutions should also try to incorporate new pedagogies and technology into training itself, such as ‘blended learning’; a method that can deliver training effectively without disturbing workflow, while leaving space for peer-to-peer support and direct training and mentorship [6, 9, 19–22].

## Conclusion

As health research institutions in Africa continue to work to maintain high quality scientific and ethical standards, particularly in the face of increasing levels of research [17], it is important to increase recognition of the critical roles played by fieldworkers, and the need to provide strong and systematic support for this group of research staff.

Through our recommendations, we envisage a system of investing in, and developing, the capacity of fieldworkers that mirrors investment in physical infrastructure, to create the capacity needed for 21^st^ century biomedical research fieldwork. We share our views on how fieldworkers’ capacity should be strengthened with the aim of building greater awareness, debate and consensus on the importance of this area of research, and ways of addressing current challenges and missed opportunities. We identify important benefits for capacity strengthening from greater collaborative efforts between African research institutions, including for planning and adequately resourcing responsive strategies at institutional levels. We also recognise the need for more research to build and evaluate context-specific approaches to strengthening fieldworker capacity in health research, an area that is relatively understudied [2, 23, 24]. The participants and research centres represented in this workshop aim to take the workshop recommendations forwards individually in their parent institutions and to develop cross-programme approaches to field worker capacity building. A second international workshop is planned in 2015 to take these ideas forwards.

### Ethical approval

The paper is published with the permission of the centre director, KEMRI. All workshop participants gave verbal consent to participate in the workshop and permitted the publication of the workshop proceedings.
